# Effects of nintedanib on circulating biomarkers of idiopathic pulmonary fibrosis

**DOI:** 10.1183/23120541.00558-2023

**Published:** 2024-12-16

**Authors:** R. Gisli Jenkins, Vincent Cottin, Yasuhiko Nishioka, Imre Noth, Eric S. White, Carina Ittrich, Claudia Diefenbach, Klaus B. Rohr, Moisés Selman, Toby M. Maher

**Affiliations:** 1 National Heart and Lung Institute, Imperial College London, London, UK; 2 National Reference Coordinating Centre for Rare Pulmonary Diseases, Louis Pradel Hospital, Hospices Civils de Lyon, Claude Bernard University Lyon 1, UMR754, Lyon, France; 3 Graduate School of Biomedical Sciences, Tokushima University, Tokushima, Japan; 4 Division of Pulmonary and Critical Care Medicine, University of Virginia, Charlottesville, VI, USA; 5 Boehringer Ingelheim Pharmaceuticals, Inc., Ridgefield, CT, USA; 6 Boehringer Ingelheim Pharma GmbH & Co. KG, Biberach an der Riss, Germany; 7 Boehringer Ingelheim International GmbH, Ingelheim am Rhein, Germany; 8 Instituto Nacional de Enfermedades Respiratorias “Ismael Cosio Villegas”, Mexico City, Mexico; 9 National Heart and Lung Institute, Imperial College London, London, UK; 10 Keck School of Medicine, University of Southern California, Los Angeles, CA, USA

## Abstract

**Background:**

Biomarkers that change in response to nintedanib in subjects with idiopathic pulmonary fibrosis (IPF) would be valuable. We investigated the effects of nintedanib on circulating biomarkers in subjects with IPF in the INMARK trial.

**Methods:**

Subjects with IPF were randomised 1:2 to receive nintedanib 150 mg twice daily or placebo for 12 weeks, after which all patients received open-label nintedanib for 40 weeks. Fold changes in adjusted mean levels of circulating biomarkers were analysed using a linear mixed model for repeated measures.

**Results:**

346 subjects were treated (116 randomised to nintedanib, 230 to placebo). Surfactant protein D (SP-D) and cancer antigen 125 (CA-125), markers of epithelial injury, decreased in subjects treated with nintedanib *versus* placebo. Fold changes from baseline in SP-D at week 12 corresponded to a 4% decrease and 3% increase in the nintedanib and placebo groups, respectively (ratio 0.94, 95% CI 0.89–0.99; p=0.024). Fold changes in CA-125 at week 12 corresponded to a 22% decrease and 4% increase in the nintedanib and placebo groups, respectively (ratio 0.75, 95% CI 0.71–0.81; p<0.0001). A mediation analysis suggested that 42.1% of the effect of nintedanib on change in forced vital capacity over 12 weeks was attributable to the change in CA-125. A small increase in C3A (collagen 3 degraded by ADAMTS-1/4/8) and a small decrease in C3M (collagen 3 degraded by matrix metalloproteinase-9), markers of extracellular matrix turnover, were observed in subjects treated with nintedanib *versus* placebo.

**Conclusions:**

Effects of nintedanib on circulating markers of epithelial dysfunction and collagen degradation, most notably CA-125, were observed in patients with IPF.

## Shareable abstract

**Data from a randomised placebo-controlled trial suggest that treatment with nintedanib has effects on circulating markers of epithelial dysfunction in patients with idiopathic pulmonary fibrosis** https://bit.ly/3LtO6vo

## Introduction

Idiopathic pulmonary fibrosis (IPF) is a progressive fibrosing interstitial lung disease (ILD) characterised by a decline in lung function and high mortality [1]. The pathogenesis of IPF is believed to be driven by epithelial cell dysfunction. The activation of epithelial cells in response to injury leads to the release of pro-fibrotic mediators, which promote the migration and proliferation of fibroblasts and their differentiation into myofibroblasts [2]. Excess deposition of extracellular matrix (ECM) proteins causes remodelling of the lung architecture, culminating in fibrosis [2].

Nintedanib is an intracellular inhibitor of tyrosine kinases that inhibits processes fundamental to the progression of pulmonary fibrosis, including fibroblast proliferation, migration and differentiation, and the deposition of ECM [3–6]. Clinical trials conducted in subjects with progressive fibrosing ILDs have shown that nintedanib reduces the rate of decline in forced vital capacity (FVC) by an average of about 50%, with a consistent effect across different types of ILD [7–12]. However, for an individual patient, there is no means of assessing response to nintedanib. Blood biomarkers that provide such information would be of great value in clinical practice.

The INMARK trial investigated blood biomarkers as predictors of disease progression and the effect of nintedanib on changes in these biomarkers in subjects with IPF and well-preserved lung function [13, 14]. The results showed no significant difference between the nintedanib and placebo groups in the rate of change in C-reactive protein degraded by matrix metalloproteinase (MMP)-1/8 (CRPM) over 12 weeks of treatment (the primary end-point), nor in the rate of change in collagen 1 degraded by MMP-2/9/13 (C1M) or collagen 3 degraded by MMP-9 (C3M). A slight increase in C-reactive protein, of which CRPM is a metabolite, was observed in the nintedanib group, compared with stable levels in the placebo group. In this analysis, we investigated the effects of nintedanib on other biomarkers of ECM turnover, epithelial dysfunction and inflammation.

## Methods

### Trial design

The design of the INMARK trial (ClinicalTrials.gov: NCT02788474) has been described previously [13, 15]. The inclusion criteria included a diagnosis of IPF according to the 2011 international guidelines [16] in the previous 3 years and FVC ≥80% predicted. Subjects were randomised 1:2 to receive nintedanib 150 mg twice daily or placebo for 12 weeks, followed by an open-label period during which all subjects received nintedanib for 40 weeks. Institutional review board or independent ethics committee approval was obtained at each site before study initiation. The INMARK trial was conducted in compliance with the protocol, the ethical principles laid down in the Declaration of Helsinki and the International Conference on Harmonisation Harmonised Tripartite Guideline for Good Clinical Practice.

### Blood sample collection and storage

Blood samples for biomarker analysis were collected at baseline and weeks 4, 8, 12, 16, 20, 24, 36 and 52. To prepare serum, blood was collected with anticoagulant-free, gel-containing serum separation tubes. Samples were allowed to clot at room temperature for approximately 60 min. Serum was separated by centrifugation and aliquoted prior to freezing. To prepare plasma, blood was collected with K2 EDTA plasma tubes that were immediately inverted 8–10 times. Plasma was separated by centrifugation and aliquoted prior to freezing. Samples were stored in a central laboratory and shipped to the sponsor or a contractor for analysis.

### Analyses

In these analyses, C3M, biglycan degraded by MMP (BGM), collagen 3 degraded by a disintegrin and metalloproteinase domain with thrombospondin motifs (ADAMTS)-1/4/8 (C3A), collagen 5 degraded by MMP-2/9 (C5M), collagen 6 degraded by MMP-2/9 (C6M), citrullinated vimentin degraded by MMP-2/8 (VICM), N-terminal propeptide of type III collagen (pro-C3), N-terminal propeptide of type VI collagen (pro-C6), lysyl oxidase-like 2 (LOXL2) and neutrophil-specific elastin fragments (NE-EL) were assessed as biomarkers of ECM turnover. Krebs von den Lungen-6 (KL-6), surfactant protein D (SP-D), cancer antigen (CA)-125 and CA19-9 were assessed as biomarkers of epithelial injury. Intercellular adhesion molecule 1 (ICAM-1) was assessed as a marker of inflammation.

Serum concentrations of each biomarker of ECM turnover were measured using an ELISA method as previously described [17]. Plasma concentrations of KL-6 and SP-D were measured using commercially available ELISA methods with minor adaptations (KL-6: Sanko Junyaku Co., Ltd./EIDIA Co., Ltd.; SP-D: BioVendor). Serum concentrations of CA-125 were measured using an electrochemiluminescence immunoassay (Beckman DxI 800). Serum concentrations of LOXL2 were measured using ELISA methods (Nordic Bioscience). Plasma concentrations of ICAM-1 and serum concentrations of CA19-9 were measured using an electrochemiluminescence immunoassay (ICAM-1: MSD; CA19-9: Roche Cobas e-601). Samples measured using ELISA were measured in duplicate with the mean of the two values used in the analyses.

Analyses were performed in subjects who received ≥1 dose of trial drug. Rates of change in the biomarkers from baseline to week 12 were analysed using a random coefficient regression model (with random slopes and intercepts) including treatment-by-time and sex, age and height as covariates. Missing data were not imputed. The baseline biomarker value was included in the response variable, together with the values at week 4, 8 and 12, rather than as an adjusting covariate, to increase the amount of data used in the estimation of each end-point. Changes from baseline in adjusted mean levels of biomarkers at each visit were based on a linear mixed model for repeated measures with fixed effects for treatment-by-visit and, for biomarkers of ECM turnover, batch. Data were not normally distributed and were log_10_ transformed before analysis. Estimates of changes from baseline were back-transformed to fold changes to aid interpretation. Analyses were not adjusted for multiplicity.

Mediation analyses were performed to determine whether the effect of nintedanib on changes in biomarkers over 12 weeks was related to the effect of nintedanib on change in FVC over 12 weeks. Models were adjusted for baseline FVC and biomarker values. Biomarkers that demonstrated an indirect relationship with FVC with p<0.1 (*i.e.*, for which there was a notable impact of change in the biomarker on change in FVC), a negative estimated slope of the regression line with p<0.05 (*i.e.*, a negative linear relationship with FVC) and a decrease with nintedanib *versus* placebo over 12 weeks with p<0.05 were included in the mediation analyses.

## Results

### Subjects

A total of 346 subjects were treated (116 randomised to nintedanib, 230 randomised to placebo). Their baseline characteristics were published previously [13]. The majority of subjects were male (76%), white (62%) and ex-smokers (69%). At baseline, mean (sd) FVC was 97.5 (13.5) % pred and mean (sd) diffusing capacity of the lung for carbon monoxide (*D*_LCO_) was 64.0 (19.8) % pred. Baseline levels of each biomarker by treatment group are shown in table 1.

**TABLE 1:** Baseline values of blood biomarkers

Biomarker	Nintedanib (n=116)^#^	Placebo (n=230)^¶^
**C3M, ng·mL^−1^**	12.1 (3.1)	12.7 (4.0)
**BGM, ng·mL^−1^**	14.0 (7.0)	15.0 (8.4)
**C3A, ng·mL^−1^**	59.4 (14.5)	58.2 (13.7)
**C5M, ng·mL^−1^**	7.4 (3.2)	6.9 (2.3)
**C6M, ng·mL^−1^**	19.9 (14.9)	21.6 (27.0)
**VICM, ng·mL^−1^**	7.5 (7.3)	6.7 (6.2)
**Pro-C3, ng·mL^−1^**	15.5 (8.8)	14.7 (6.6)
**Pro-C6, ng·mL^−1^**	10.2 (6.4)	9.4 (5.0)
**LOXL-2, ng·mL^−1^**	187.8 (142.4)	182.9 (139.4)
**NE-EL, ng·mL^−1^**	8.9 (5.7)	9.7 (8.3)
**KL-6, U·mL^−1^**	1144.2 (983.5)	1087.5 (760.8)
**SP-D, ng·mL^−1^**	725.6 (556.3)	690.2 (441.2)
**CA-125, U·mL^−1^**	14.9 (8.8)	15.1 (10.2)
**CA19-9, U·mL^−1^**	19.5 (26.3)	34.1 (95.8)
**ICAM-1, ng·mL^−1^**	649.3 (164.5)	651.0 (180.1)

Data are presented as mean (sd). All biomarkers were measured in serum except for Krebs von den Lungen-6 (KL-6), surfactant protein D (SP-D) and intercellular adhesion molecule 1 (ICAM-1), which were measured in plasma. ^#^: n=115 for citrullinated vimentin degraded by matrix metalloproteinase (MMP)-2/8 (VICM), neutrophil-specific elastin fragments (NE-EL), SP-D and ICAM-1; n=110 for N-terminal propeptide of type III collagen (pro-C3) and N-terminal propeptide of type VI collagen (pro-C6); n=81 for lysyl oxidase-like 2 (LOXL-2); n=68 for cancer antigen (CA)-125; n=65 for CA19-9. ^¶^: n=229 for KL-6; n=228 for collagen 3 degraded by MMP-9 (C3M), collagen 3 degraded by a disintegrin and metalloproteinase domain with thrombospondin motifs (ADAMTS)-1/4/8 (C3A), VICM, SP-D and ICAM-1; n=226 for biglycan degraded by MMP (BGM), collagen 5 degraded by MMP-2/9 (C5M) and NE-EL; n=225 for collagen 6 degraded by MMP-2/9 (C6M); n=220 for pro-C3; n=218 for pro-C6; n=170 for LOXL-2; n=154 for CA-125; n=141 for CA19-9.

### Markers of epithelial injury

**FIGURE 1:**
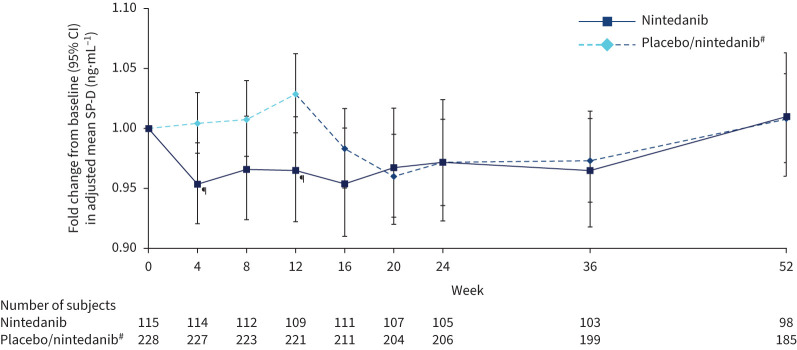
Fold changes from baseline in surfactant protein D (SP-D) over 52 weeks. ^#^: Subjects received placebo (blinded) for 12 weeks followed by nintedanib (open label) for 40 weeks. ^¶^: p<0.05 for adjusted difference in change from baseline between groups.

There was a significant reduction in change in SP-D over 12 weeks with nintedanib *versus* placebo. The adjusted mean (se) rate of change in SP-D over 12 weeks was −4.91 (3.70)×10^−3^ ng·mL^−1^·month^−1^ in the nintedanib group and 4.08 (2.62)×10^−3^ ng·mL^−1^·month^−1^ in the placebo group (difference −8.99 (95% CI −17.89 – −0.10)×10^−3^ ng·mL^−1^·month^−1^; p=0.047). Fold changes from baseline in adjusted mean SP-D at week 12 were 0.96 and 1.03 in the nintedanib and placebo groups, respectively (ratio 0.94, 95% CI 0.89–0.99); p=0.024). The difference in fold change from baseline in SP-D with nintedanib *versus* placebo was apparent from week 4 (figure 1). From week 12 to week 16, there was a reduction in SP-D in subjects initially treated with placebo who started nintedanib at week 12 (ratio for week 16 *versus* week 12: 0.96, 95% CI 0.93–0.98; p=0.0011). After week 16, the fold change from baseline was similar between groups.

**FIGURE 2:**
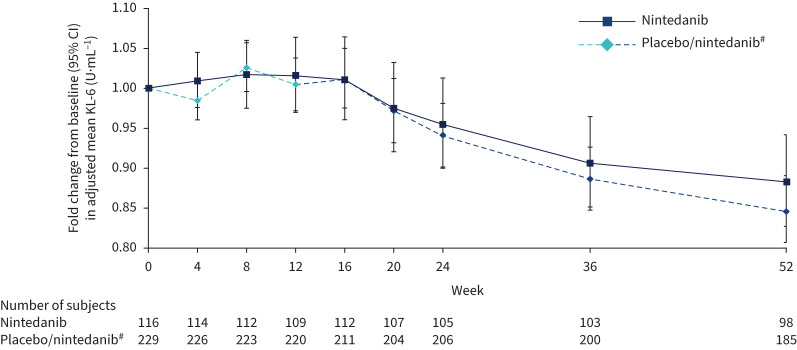
Fold changes from baseline in Krebs von den Lungen-6 (KL-6) over 52 weeks. ^#^: Subjects received placebo (blinded) for 12 weeks followed by nintedanib (open label) for 40 weeks.

There was no significant difference in change in KL-6 over 12 weeks with nintedanib *versus* placebo. The adjusted mean (se) rate of change in KL-6 over 12 weeks was 2.40 (3.73)×10^−3^ U·mL^−1^·month^−1^ in the nintedanib group and 2.72 (2.65)×10^−3^ U·mL^−1^·month^−1^ in the placebo group (difference −0.32 (95% CI −9.28–8.64)×10^−3^ U·mL^−1^·month^−1^; p=0.94). Fold changes from baseline in adjusted mean KL-6 at week 12 were 1.02 and 1.00 in the nintedanib and placebo groups, respectively (ratio 1.01, 95% CI 0.96–1.07; p=0.69). Although the fold change from baseline in KL-6 at week 12 was similar between nintedanib and placebo, KL-6 appeared to decrease with prolonged treatment (ratios for week 52 *versus* week 12: 0.87 (95% CI 0.82–0.92; p<0.0001) and 0.84 (95% CI 0.81–0.88; p<0.0001) in the nintedanib and placebo groups, respectively) (figure 2).

**FIGURE 3:**
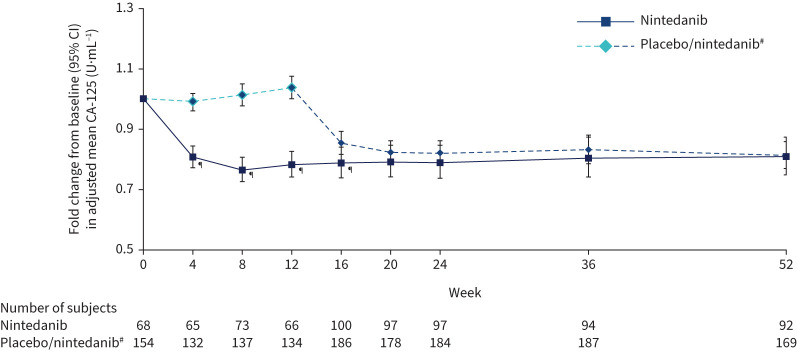
Fold changes from baseline in cancer antigen 125 (CA-125) over 52 weeks. ^#^: Subjects received placebo (blinded) for 12 weeks followed by nintedanib (open label) for 40 weeks. ^¶^: p<0.05 for adjusted difference in change from baseline between groups.

There was a significant reduction in change in CA-125 over 12 weeks with nintedanib *versus* placebo. The adjusted mean (se) rate of change in CA-125 over 12 weeks was −38.77 (4.43)×10^−3^ U·mL^−1^·month^−1^ in the nintedanib group and 7.18 (3.02)×10^−3^ U·mL^−1^·month^−1^ in the placebo group (difference −45.95 (95% CI −56.45 – −35.46)×10^−3^ U·mL^−1^·month^−1^; p<0.0001). Fold changes in adjusted mean CA-125 at week 12 were 0.78 and 1.04 in the nintedanib and placebo groups, respectively (ratio 0.75, 95% CI 0.71–0.81; p<0.0001). Fold changes in CA-125 decreased with nintedanib *versus* placebo from week 4. From week 12 to week 16, there was a similar reduction in CA-125 in subjects initially treated with placebo who started nintedanib at week 12 (ratio for week 16 *versus* week 12: 0.82, 95% CI 0.80–0.85; p<0.0001). After week 16, CA-125 levels were similar between the groups (figure 3).

There was no significant difference between nintedanib and placebo in change in CA19-9 over 12 weeks (see table E1 in the online data supplement). Fold changes from baseline in CA19-9 over 52 weeks were similar in both groups (see figure E1 in the online data supplement).

### Markers of ECM turnover

**FIGURE 4:**
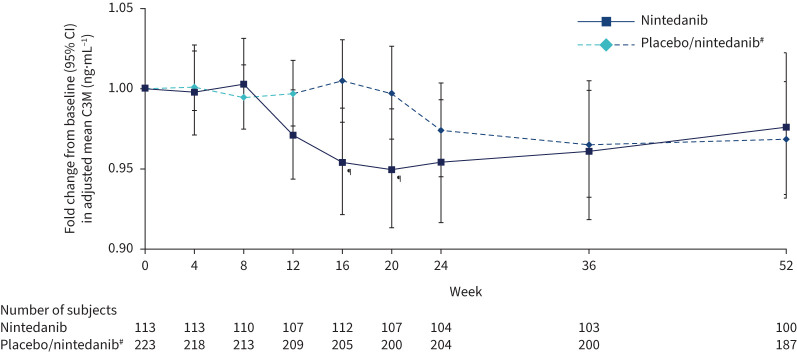
Fold changes from baseline in collagen 3 degraded by MMP-9 (C3M) over 52 weeks. ^#^: Subjects received placebo (blinded) for 12 weeks followed by nintedanib (open label) for 40 weeks. ^¶^: p<0.05 for adjusted difference in change from baseline between groups.

Nonsignificant differences were observed in the adjusted rate of changes in C3M and C3A over 12 weeks in subjects treated with nintedanib compared with placebo [13]. Fold changes from baseline in adjusted mean C3M at week 12 were 0.97 and 1.00 in the nintedanib and placebo groups, respectively (ratio 0.97, 95% CI 0.94–1.01; p=0.14). A decrease in the fold change from baseline in C3M was evident after 12 weeks of treatment with nintedanib *versus* placebo, with significant ratios at weeks 16 and 20 (figure 4). A decrease in C3M was observed after week 24 in subjects treated with placebo for 12 weeks followed by nintedanib, similar to the decrease observed after week 12 in subjects randomised to nintedanib.

**FIGURE 5:**
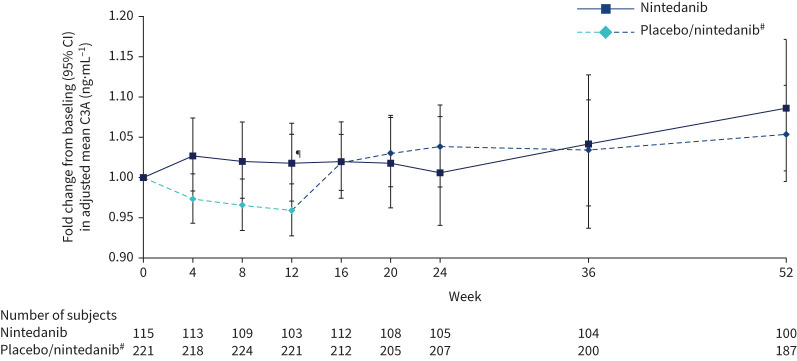
Fold changes from baseline in collagen 3 degraded by ADAMTS-1/4/8 (C3A) over 52 weeks. ^#^: Subjects received placebo (blinded) for 12 weeks followed by nintedanib (open label) for 40 weeks. ^¶^: p<0.05 for adjusted difference in change from baseline between groups.

The adjusted mean (se) rate of change in C3A over 12 weeks was 1.76 (3.50)×10^−3^ ng·mL^−1^·month^−1^ in the nintedanib group and −4.97 (2.50)×10^−3^ ng·mL^−1^·month^−1^ in the placebo group (difference 6.73 (95% CI −1.64–15.10)×10^−3^ ng·mL^−1^·month^−1^; p=0.11). Fold changes from baseline in adjusted mean C3A at week 12 were 1.02 and 0.96 in the nintedanib and placebo groups, respectively (ratio 1.06, 95% CI 1.00–1.12; p=0.046). An increase in the fold change from baseline in C3A with nintedanib *versus* placebo was apparent from week 4 to week 12. After week 12, the fold change from baseline was similar between groups. The increase in C3A between weeks 12 and 16 in subjects treated with placebo for 12 weeks followed by nintedanib was similar to the increase observed between baseline and week 4 in subjects randomised to nintedanib (figure 5).

The adjusted mean (se) rate of change in VICM over 12 weeks was −19.81×10^−3^ (9.54×10^−3^) ng·mL^−1^·month^−1^ in the nintedanib group and 4.81×10^−3^ (6.94×10^−3^) ng·mL^−1^·month^−1^ in the placebo group (difference −24.63 (95% CI −46.94 – −2.31)×10^−3^ ng·mL^−1^·month^−1^; p=0.031). Fold changes from baseline in VICM are shown in figure E2. An increase in the fold change from baseline in pro-C3 with nintedanib *versus* placebo was apparent at week 4 (figure E3). After week 4, the fold change from baseline was similar between groups. A decrease in the fold change from baseline in pro-C6 with nintedanib *versus* placebo was apparent from week 4 to week 12 (figure E4). After week 12, the fold change from baseline was similar between groups.

For the other markers of ECM turnover, no notable trends were observed in the adjusted mean (se) rates of change over 12 weeks (see table E1 in the online data supplement) or in fold changes over 52 weeks (see figures E5–9 in the online data supplement).

### Marker of inflammation

**FIGURE 6:**
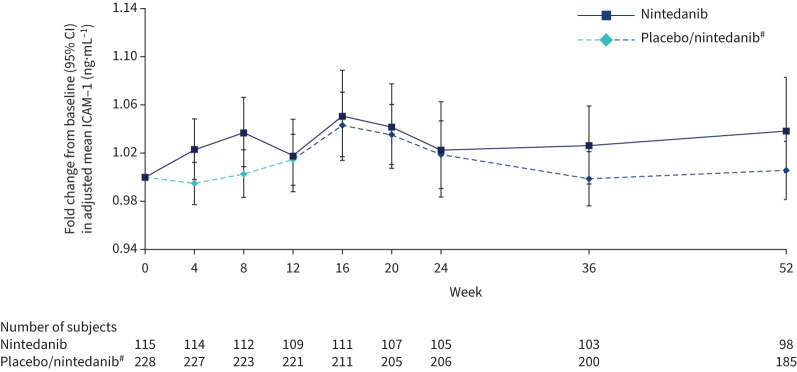
Fold changes from baseline in intercellular adhesion molecule 1 (ICAM-1) over 52 weeks. ^#^: Subjects received placebo (blinded) for 12 weeks followed by nintedanib (open label) for 40 weeks.

There was no significant difference in change in ICAM-1 over 12 weeks with nintedanib *versus* placebo. The adjusted mean (se) rate of change in ICAM-1 over 12 weeks was 3.25 (2.33)×10^−3^ ng·mL^−1^·month^−1^ in the nintedanib group and 2.16 (1.65)×10^−3^ ng·mL^−1^·month^−1^ in the placebo group (difference 1.09 (95% CI −4.51–6.68)×10^−3^ ng·mL^−1^·month^−1^; p=0.70). Fold changes in adjusted mean ICAM-1 at week 12 were 1.02 and 1.01 in the nintedanib and placebo groups, respectively (ratio 1.00, 95% CI 0.97–1.04); p=0.86). Fold changes from baseline in ICAM-1 over 52 weeks were similar in both groups (figure 6).

### Mediation analyses

**FIGURE 7:**
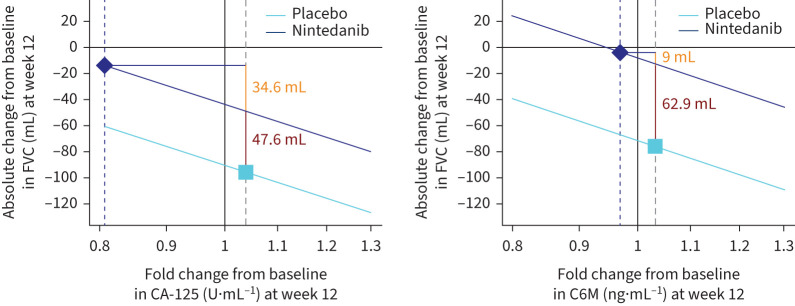
Mediation analysis of change in forced vital capacity (FVC) attributable to changes in cancer antigen 125 (CA-125) and collagen 6 degraded by matrix metalloproteinase-2/9 (C6M) at week 12. The yellow values denote the indirect effects that the change in the biomarker has on the change in FVC (mL). The red values denote the direct effect of nintedanib on the change in FVC (mL) not mediated by the change in the biomarker.

Scatter plots depicting the correlation between changes from baseline in CA-125 at week 12 and changes from baseline in FVC at week 12 in the nintedanib and placebo groups are shown in figure E10. Two biomarkers met the criteria for the mediation analysis, namely CA-125 and C6M (figure 7). In the analysis of CA-125, the total treatment effect of nintedanib on change in FVC at week 12 was 82.2 mL; of this, a change of 34.6 mL (42.1%) was attributed to the change in CA-125. In the analysis of C6M, the total treatment effect of nintedanib on change in FVC at week 12 was 72.0 mL; of this, a change of 9.0 mL (12.5%) was attributed to the change in C6M.

## Discussion

The INMARK trial provided a rare opportunity to investigate the effects of antifibrotic therapy on biomarkers of epithelial injury, ECM turnover and inflammation in subjects with IPF in a randomised placebo-controlled setting. Data from the first 12 weeks of the open-label period, in which subjects initially treated with placebo started taking nintedanib, provided an opportunity to confirm the observations seen in patients randomised to nintedanib. Our data suggest that nintedanib may have effects on circulating levels of SP-D and CA-125, markers of epithelial dysfunction, a critical and early event in the pathogenesis of IPF [2], over as little as 4 weeks, although it cannot be determined from these data whether nintedanib has a direct effect on the epithelium. These findings are consistent with a retrospective analysis of data from 56 patients with IPF, which showed that levels of SP-D and CA-125 significantly increased over 1 year in untreated subjects with IPF, but decreased in subjects treated with nintedanib [18]. In our analysis, the greatest effect of nintedanib was observed on CA-125; changes in the other biomarkers measured were small. In a mediation analysis performed to explore the association between changes in CA-125 and changes in FVC, about 40% of the effect of nintedanib on change in FVC over 12 weeks could be attributed to treatment-related changes in CA-125. Thus, about 60% of the effect of nintedanib on change in FVC over 12 weeks was independent of CA-125.

In our study, nintedanib did not markedly reduce KL-6 over 12 weeks, but KL-6 may be reduced with more prolonged treatment. Uncontrolled studies of KL-6 in treated and untreated patients with IPF have generated variable results [19–25]. Among 66 untreated patients with IPF followed for a median of 49.5 months, 43.9, 31.8 and 24.2% had increased, decreased and unchanged KL-6 levels from baseline [20]. A prospective study in 23 subjects found no change in KL-6 levels after 12 months of nintedanib treatment [22]. In another prospective study of 26 subjects receiving nintedanib, KL-6 concentrations increased over 6 months in subjects who had a relative decline in FVC ≥10% or a relative decline in *D*_LCO_ ≥15%, but decreased in other subjects [23]. In a multivariable analysis of data from 62 subjects with IPF who were treated with antifibrotic therapy and had baseline KL-6 levels ≥500 U·mL^−1^, higher relative changes in KL-6 levels over 1 month were associated with disease progression (relative decline in FVC % pred ≥10% or *D*_LCO_ % pred ≥15%, acute exacerbation or death within 6 months of starting antifibrotic therapy) [25].

Nintedanib has been shown to reduce pro-collagen 1 mRNA and total collagen in lung tissue in bleomycin models of epithelial cell injury-induced lung inflammation and fibrosis [4, 5], to reduce transforming growth factor-β1-induced elevations in N-terminal propeptide of type I collagen (pro-C1), pro-C3, pro-C6 and fibronectin in primary lung fibroblasts from healthy donors [26] and to reduce pro-C3 and C3M in precision-cut lung slices from subjects with pulmonary fibrosis [27]. In these analyses of data from the INMARK trial, small changes in C6M were observed, which were inconsistent over time, and the mediation analysis suggested that change in C6M made only a small contribution to the relationship between nintedanib treatment and change in FVC. Small increases in C3A and small decreases in C3M, VICM and pro-C6 were observed over 12 weeks in subjects treated with nintedanib *versus* placebo, with the effects on C3A seen earlier than the effects on C3M. Data from observational studies have suggested that levels of VICM, pro-C3 and pro-C6 are elevated in subjects with IPF [28–30]. In the INMARK trial, no notable trends were observed in subjects treated with nintedanib compared with placebo for the other biomarkers of ECM turnover analysed or for ICAM-1, a biomarker of inflammation.

Strengths of our study include the prospective design that included a placebo-controlled period and an internal validation period after subjects who initially received placebo were switched to nintedanib, as well as the use of flexible mixed models for repeated measures to assess changes in biomarkers over time. Our study also has limitations. Assays for markers of ECM turnover required batch correction. The patients included in the INMARK trial had well-preserved lung function and it is possible that biomarker levels, and the effects of nintedanib on biomarker levels, may not be the same in subjects with early compared with more advanced IPF. The randomised double-blind period was limited to 12 weeks and it may be that a longer treatment period is required to see an effect of nintedanib on specific circulating biomarkers.

In conclusion, data from the INMARK trial suggest that in subjects with IPF and preserved FVC, nintedanib may have early and significant effects on circulating levels of CA-125 and, to a lesser extent, effects on SP-D, C3A and C3M. Further studies are needed to explore the mechanisms by which nintedanib reduces CA-125 and to establish if CA-125 is a clinically relevant marker of response to nintedanib.

## Supplementary material

10.1183/23120541.00558-2023.Supp1 **Please note:** supplementary material is not edited by the Editorial Office, and is uploaded as it has been supplied by the author.
Supplementary material 00558-2023.SUPPLEMENT

## Data Availability

To ensure independent interpretation of clinical study results and enable authors to fulfil their role and obligations under the International Committee of Medical Journal Editors criteria, Boehringer Ingelheim grants all external authors access to relevant clinical study data. In adherence with the Boehringer Ingelheim Policy on Transparency and Publication of Clinical Study Data, scientific and medical researchers can request access to clinical study data after publication of the primary manuscript in a peer-reviewed journal, regulatory activities are complete and other criteria are met. Researchers should use https://vivli.org/ to request access to study data and visit https://www.mystudywindow.com/msw/datasharing for further information.
