# Supplementary material for: Effects of nintedanib on circulating biomarkers of idiopathic pulmonary fibrosis
Source: ERJ Open Res. 2024 Dec 16;10(6):00558-2023. doi: 10.1183/23120541.00558-2023 (PMC11647937; doi:10.1183/23120541.00558-2023)
Supplement: Supplementary file 1 [file 00558-2023.SUPPLEMENT.pdf]

Effects of nintedanib on circulating biomarkers of idiopathic pulmonary fibrosis

Online Data Supplement

Table E1: Adjusted mean (SE) rates of change in BGM, C5M, C6M, VICM, pro-C3, pro-C6, LOXL2, NE-EL, and CA19-9 over 12 weeks

	**Nintedanib (n=116)***	**Placebo (n=229)†**
Adjusted rate (SE) of change in BGM from baseline to week 12	7.90×10^{-3} (5.05×10^{-3})	4.13×10^{-3} (3.67×10^{-3})
Difference (95% CI)	3.77×10^{-3} (-8.08×10^{-3}, 15.63×10^{-3})	
p-value	0.53	
Adjusted rate (SE) of change in C5M from baseline to week 12	-1.74×10^{-3} (3.53×10^{-3})	-1.53×10^{-3} (2.53×10^{-3})
Difference (95% CI)	-0.21×10^{-3} (-8.65×10^{-3}, 8.22×10^{-3})	
p-value	0.96	
Adjusted rate (SE) of change in C6M from baseline to week 12	-5.40×10^{-3} (5.49×10^{-3})	-3.72×10^{-3} (4.01×10^{-3})
Difference (95% CI)	-1.68×10^{-3} (-14.52×10^{-3}, 11.16×10^{-3})	
p-value	0.80	
Adjusted rate (SE) of change in VICM from baseline to week 12	-19.81×10^{-3} (9.54×10^{-3})	4.81×10^{-3} (6.94×10^{-3})
Difference (95% CI)	-24.63 (-46.94×10^{-3}, -2.31×10^{-3})	

p-value	0.031	
Adjusted rate (SE) of change in pro-C3 from baseline to week 12	2.24×10^{-3} (4.27×10^{-3})	-1.62×10^{-3} (3.08×10^{-3})
Difference (95% CI)	3.86×10^{-3} (-6.25×10^{-3}, 13.97×10^{-3})	
p-value	0.45	
Adjusted rate (SE) of change in pro-C6 from baseline to week 12	-9.37×10^{-3} (4.78×10^{-3})	0.86×10^{-3} (3.53×10^{-3})
Difference (95% CI)	-10.22×10^{-3} (-21.41×10^{-3}, 0.96×10^{-3})	
p-value	0.073	
Adjusted rate (SE) of change in LOXL2 from baseline to week 12	8.13×10^{-3} (6.99×10^{-3})	3.91×10^{-3} (5.04×10^{-3})
Difference (95% CI)	4.22×10^{-3} (-12.08×10^{-3}, 20.53×10^{-3})	
p-value	0.61	
Adjusted rate (SE) of change in NE-EL from baseline to week 12	8.82×10^{-3} (7.38×10^{-3})	9.76×10^{-3} (5.41×10^{-3})
Difference (95% CI)	-0.94×10^{-3} (-18.07×10^{-3}, 16.20×10^{-3})	
p-value	0.91	
Adjusted rate (SE) of change in CA19-9 from baseline to week 12	-12.78×10^{-3} (13.63×10^{-3})	9.77×10^{-3} (9.65×10^{-3})
Difference (95% CI)	-22.55×10^{-3} (-54.53×10^{-3}, 9.43×10^{-3})	
p-value	0.17	

*n=115 for pro-C3, pro-C6; n=99 for CA19-9; n=97 for LOXL2. †n=229 for pro-C3; n=228 for C5M, pro-C6, NE-EL; n=193 for LOXL2; n=190 for CA19-9.

Figure E1. Fold changes from baseline in CA19-9 over 52 weeks

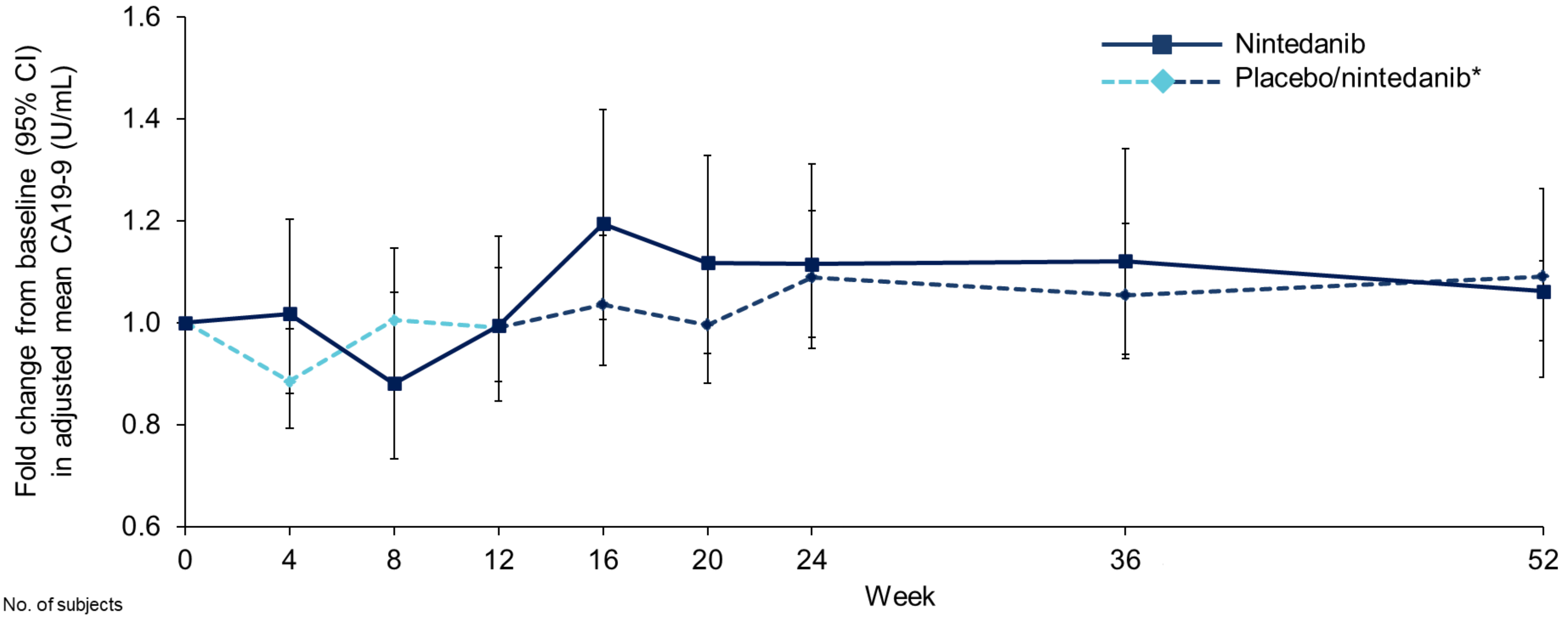

No. of subjects									
Nintedanib	65	63	64	60	97	93	95	89	85
Placebo/nintedanib*	141	135	121	117	172	173	174	175	161

*Subjects received placebo (blinded) for 12 weeks followed by nintedanib (open-label) for 40 weeks.

Figure E2. Fold changes from baseline in VICM over 52 weeks

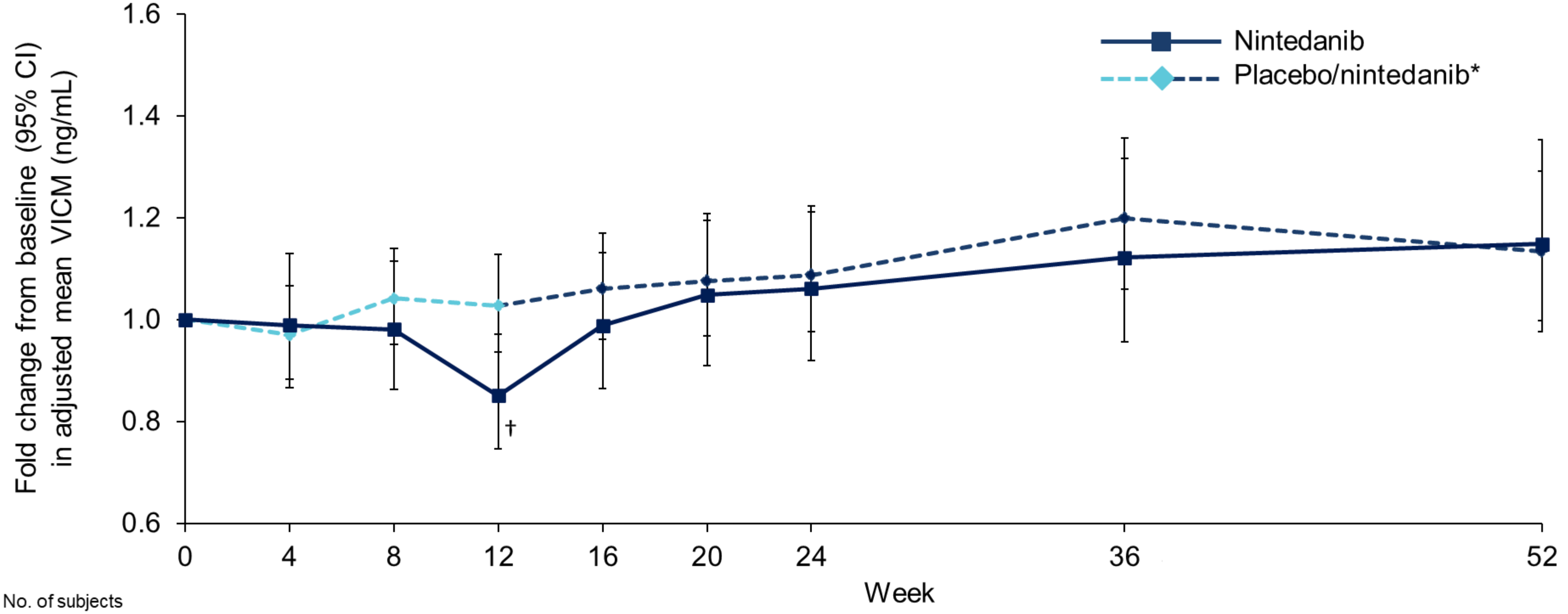

No. of subjects	0	4	8	12	16	20	24	36	52
Nintedanib	115	115	113	110	112	108	104	104	100
Placebo/nintedanib*	228	227	224	219	208	204	207	200	187

*Subjects received placebo (blinded) for 12 weeks followed by nintedanib (open-label) for 40 weeks.

†$p<0.05$ for adjusted difference in change from baseline between groups.

Figure E3. Fold changes from baseline in pro-C3 over 52 weeks

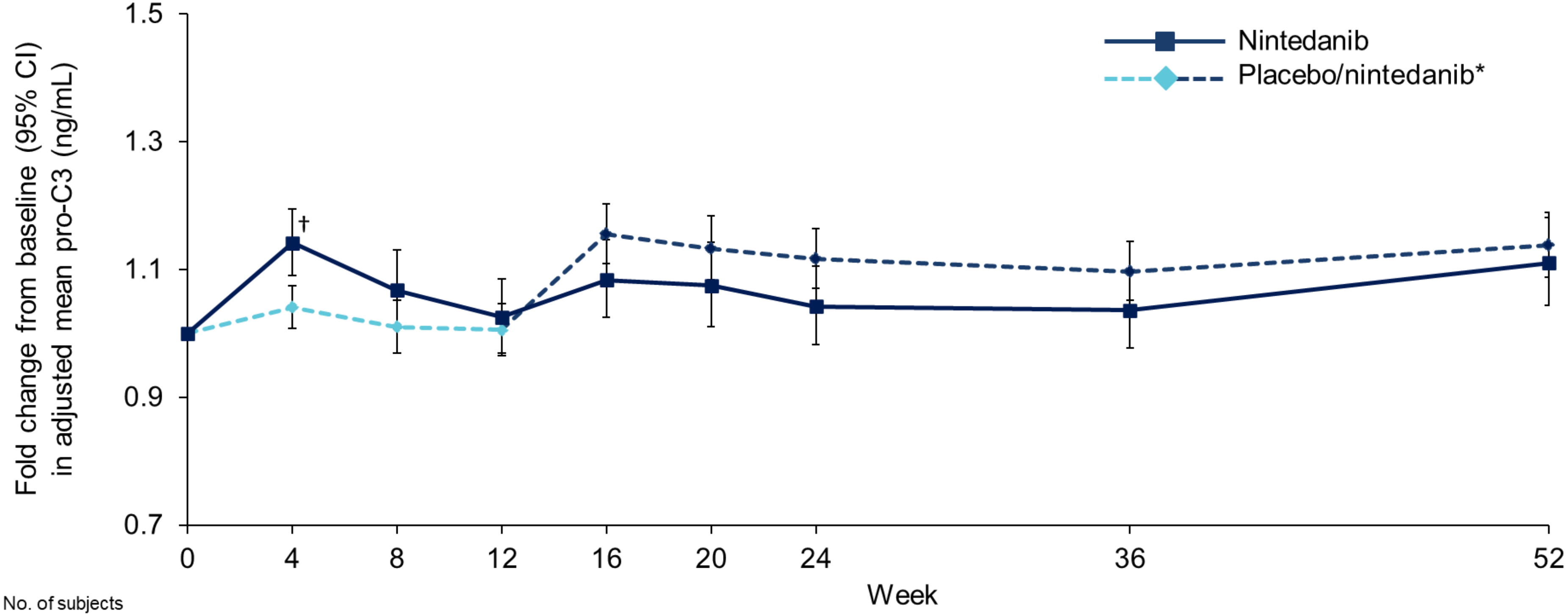

No. of subjects									
Nintedanib	110	111	112	107	109	104	101	103	99
Placebo/nintedanib*	217	217	217	208	203	202	205	199	186

*Subjects received placebo (blinded) for 12 weeks followed by nintedanib (open-label) for 40 weeks.
†p<0.05 for adjusted difference in change from baseline between groups.

Figure E4. Fold changes from baseline in pro-C6 over 52 weeks

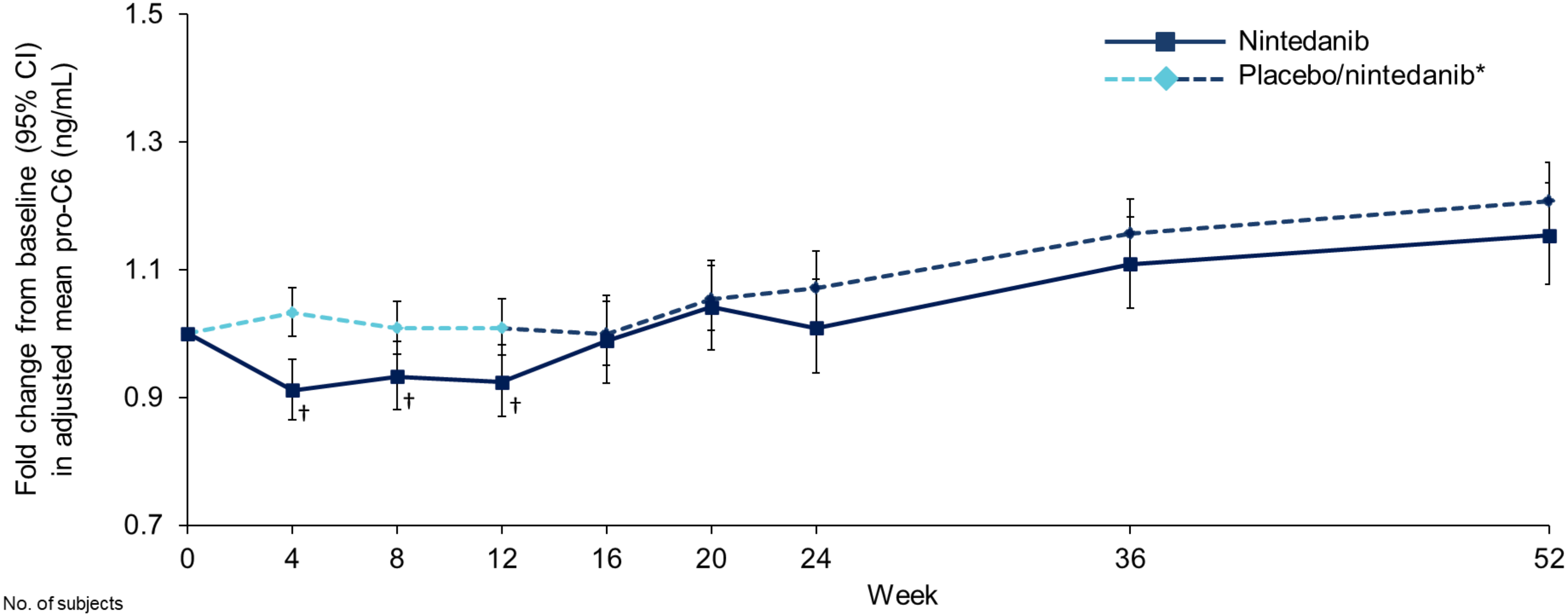

No. of subjects									
Nintedanib	110	111	111	109	110	106	105	103	100
Placebo/nintedanib*	218	216	217	206	208	205	206	200	186

*Subjects received placebo (blinded) for 12 weeks followed by nintedanib (open-label) for 40 weeks.
†p<0.05 for adjusted difference in change from baseline between groups.

Figure E5. Fold changes from baseline in BGM over 52 weeks

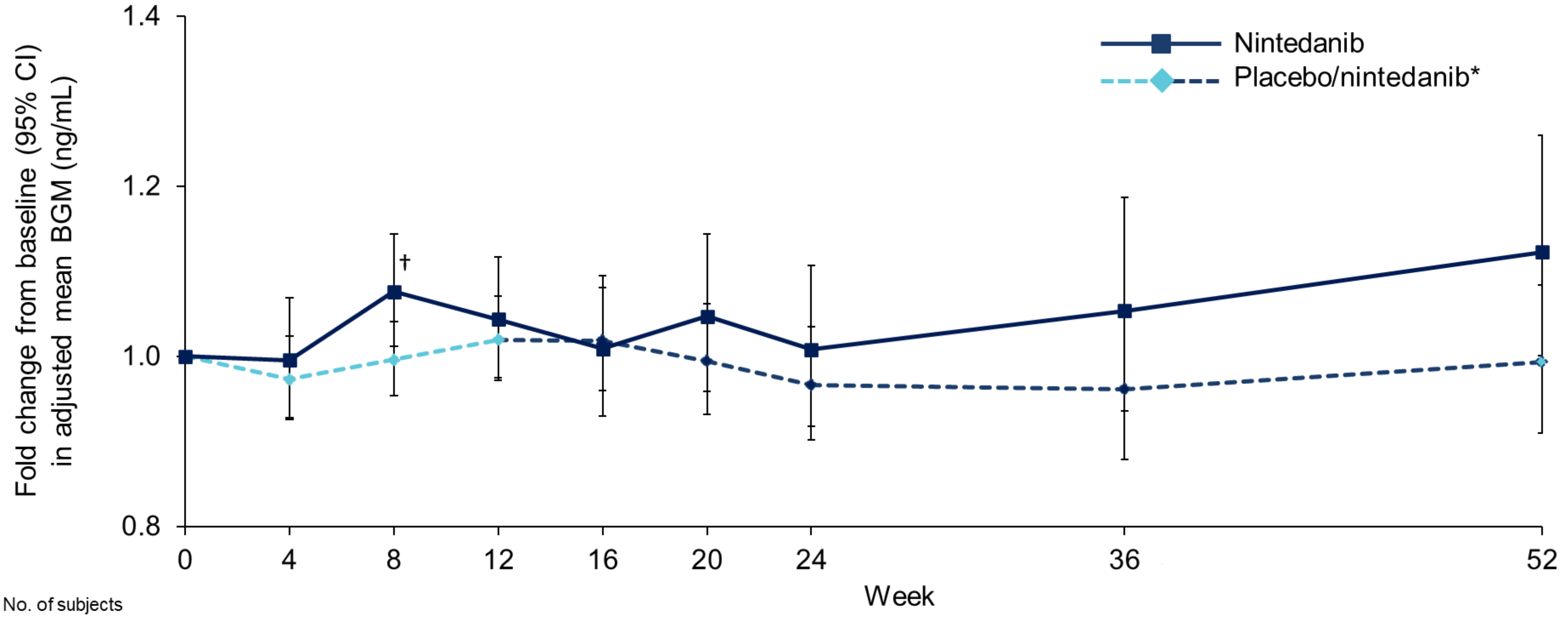

No. of subjects									
Nintedanib	116	114	112	110	102	91	85	80	73
Placebo/nintedanib*	226	225	221	220	190	181	174	160	150

*Subjects received placebo (blinded) for 12 weeks followed by nintedanib (open-label) for 40 weeks.
†p<0.05 for adjusted difference in change from baseline between groups.

Figure E6. Fold changes from baseline in C5M over 52 weeks

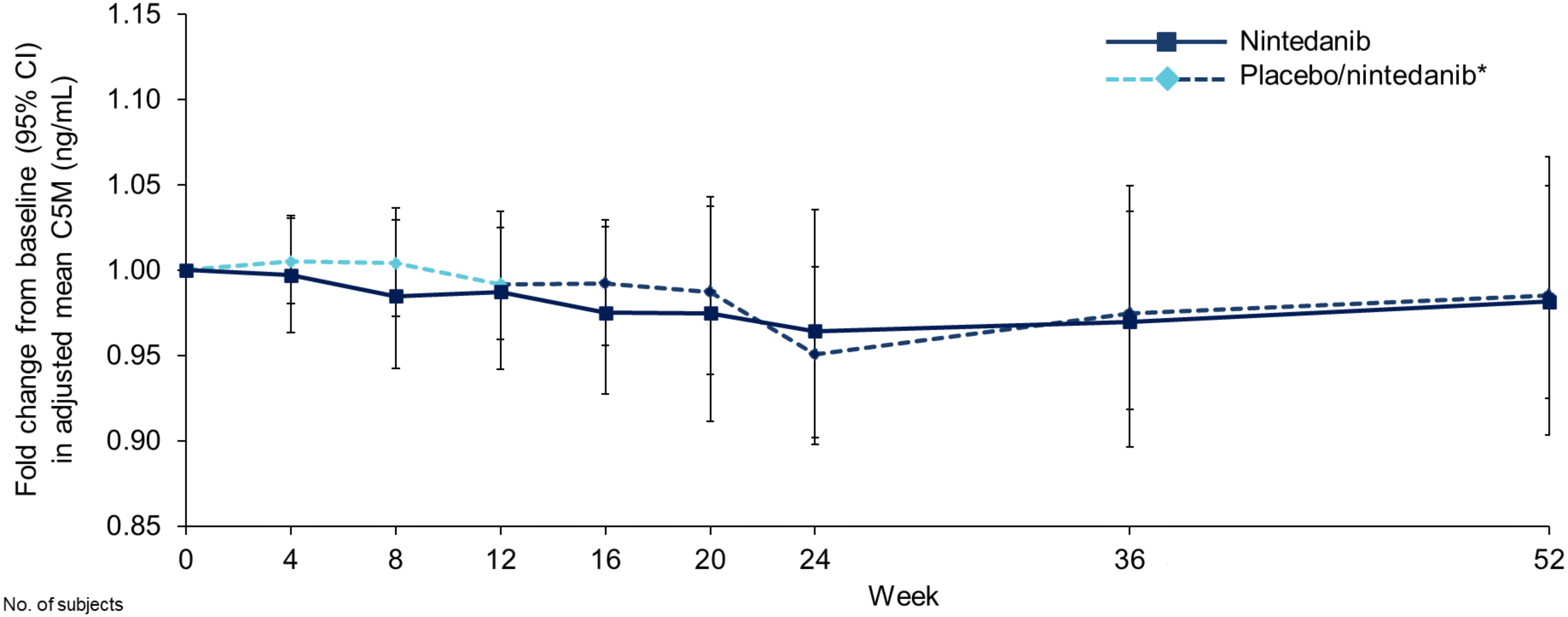

No. of subjects	0	4	8	12	16	20	24	36	52
Nintedanib	116	114	112	110	112	108	105	104	100
Placebo/nintedanib*	225	224	221	218	209	204	207	200	187

*Subjects received placebo (blinded) for 12 weeks followed by nintedanib (open-label) for 40 weeks.

Figure E7. Fold changes from baseline in C6M over 52 weeks

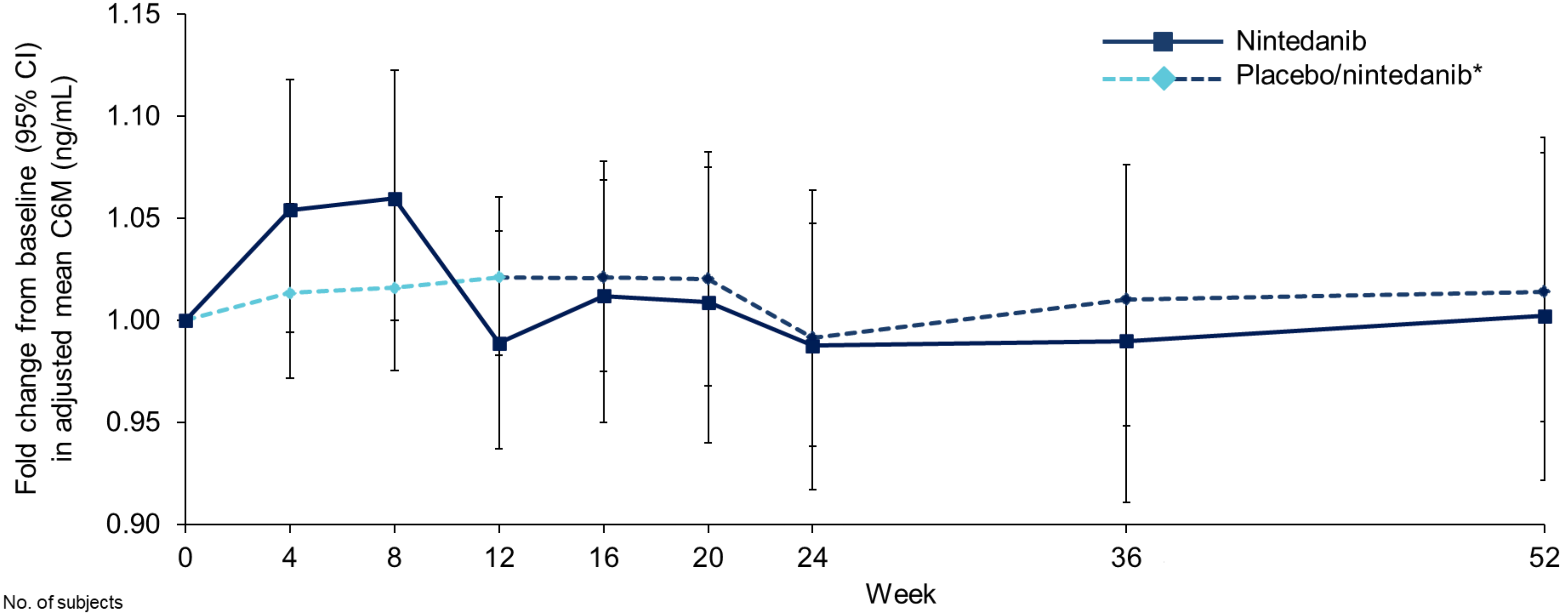

No. of subjects									
Nintedanib	111	109	103	99	110	106	103	103	100
Placebo/nintedanib*	218	212	208	203	206	202	206	200	186

*Subjects received placebo (blinded) for 12 weeks followed by nintedanib (open-label) for 40 weeks.

Figure E8. Fold changes from baseline in LOXL2 over 52 weeks

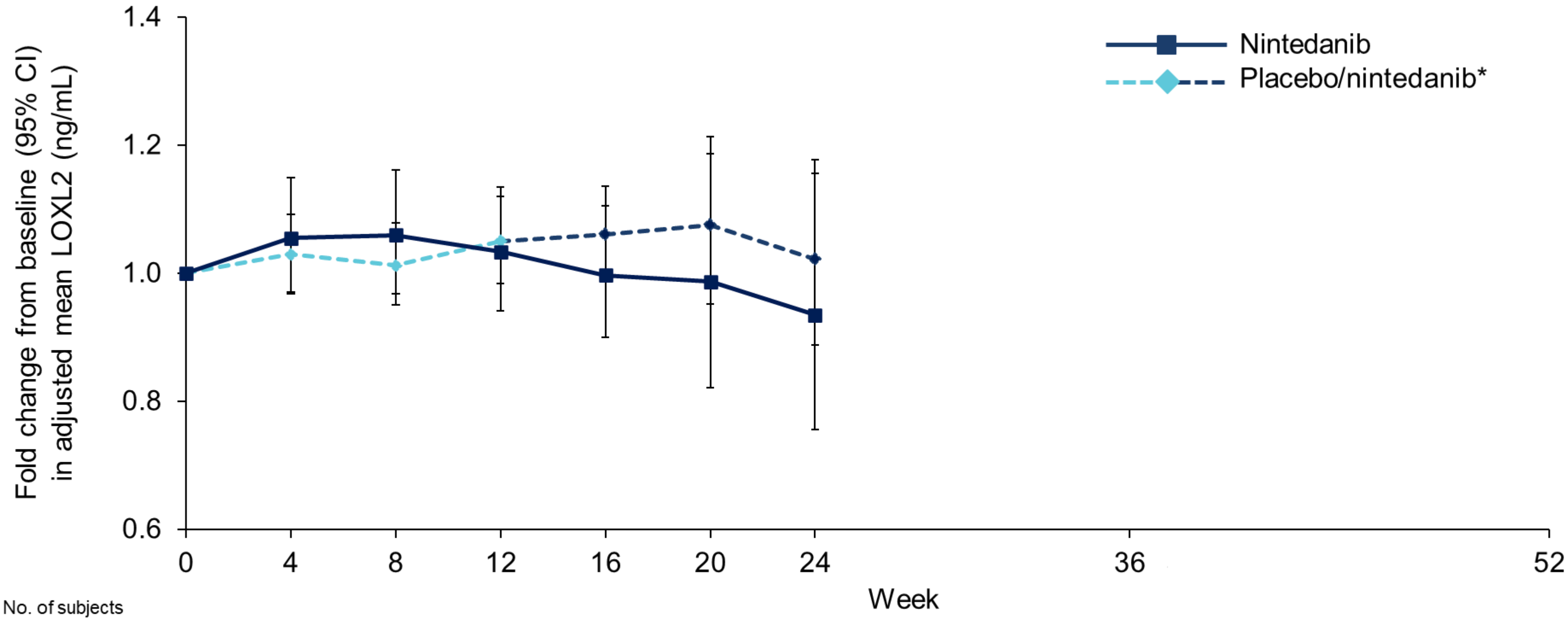

No. of subjects							
Nintedanib	81	82	84	77	53	36	27
Placebo/nintedanib*	170	170	167	156	119	85	63

*Subjects received placebo (blinded) for 12 weeks followed by nintedanib (open-label) for 40 weeks.
After week 24, data were unavailable due to exhaustion of samples.

Figure E9. Fold changes from baseline in NE-EL over 52 weeks

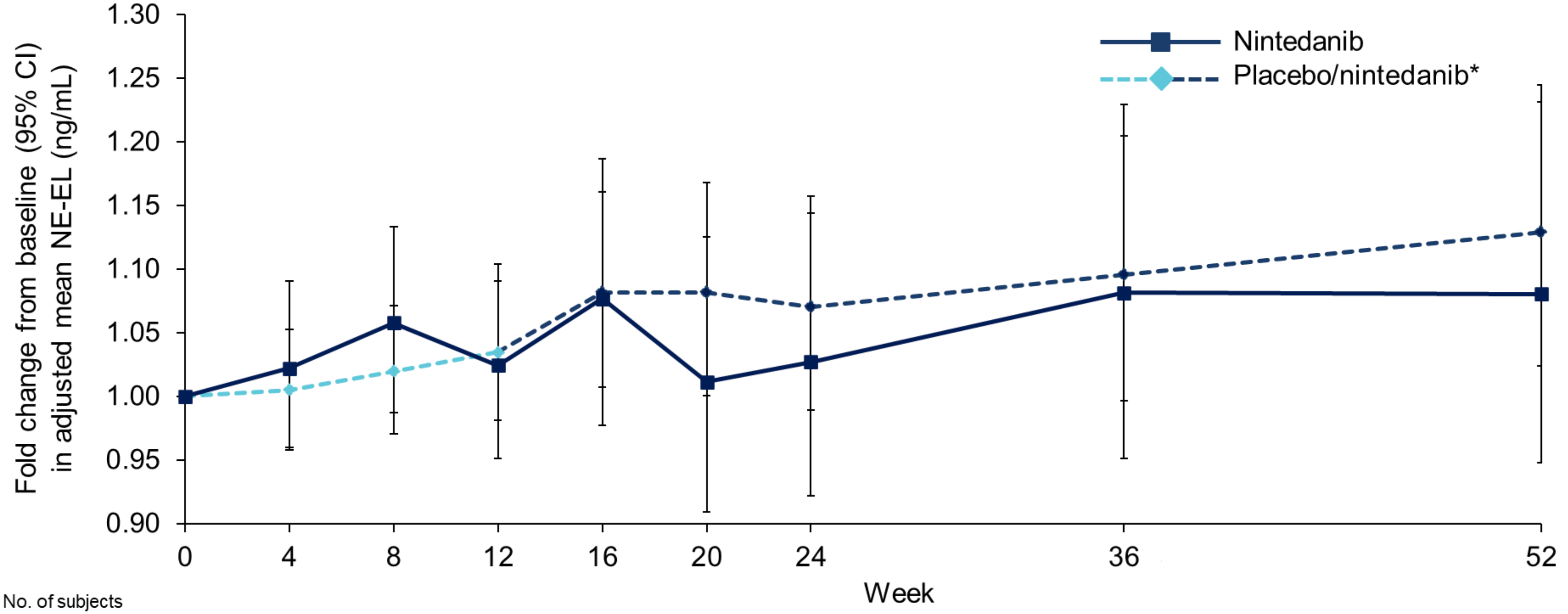

No. of subjects									
Nintedanib	115	114	111	108	110	107	105	104	100
Placebo/nintedanib*	226	225	220	216	205	204	206	199	187

*Subjects received placebo (blinded) for 12 weeks followed by nintedanib (open-label) for 40 weeks.

Figure E10. Correlation between changes from baseline in CA-125 at week 12 and changes from baseline in FVC at week 12 in the nintedanib and placebo groups

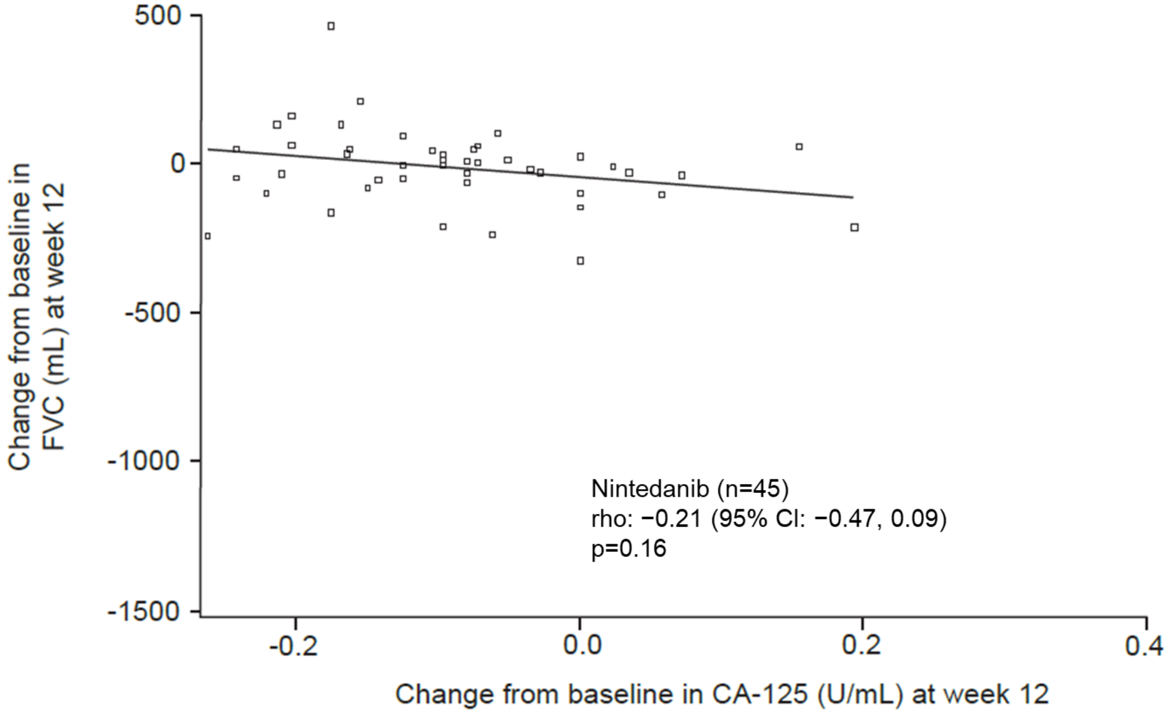

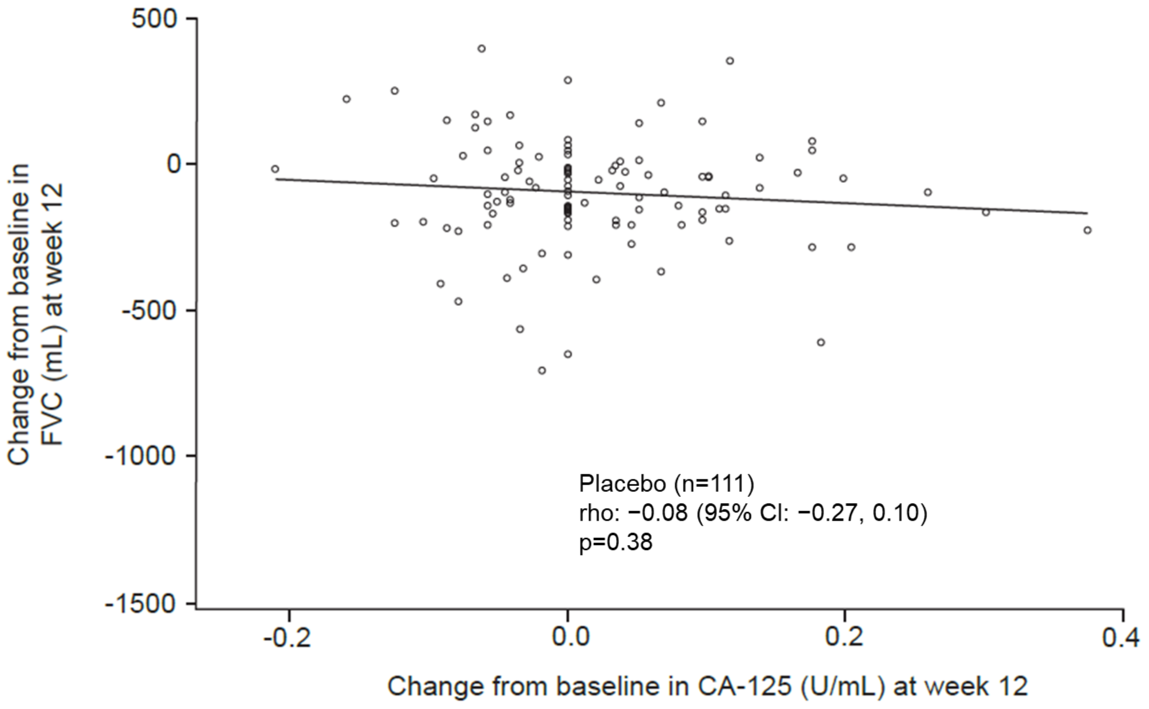

CA-125 values were log_{10} transformed.

Data sharing statement

To ensure independent interpretation of clinical study results and enable authors to fulfill their role and obligations under the ICMJE criteria, Boehringer Ingelheim grants all external authors access to relevant clinical study data. In adherence with the Boehringer Ingelheim Policy on Transparency and Publication of Clinical Study Data, scientific and medical researchers can request access to clinical study data after publication of the primary manuscript in a peer-reviewed journal, regulatory activities are complete and other criteria are met. Researchers should use https://vivli.org/ to request access to study data and visit https://www.mystudywindow.com/msw/datasharing for further information.
